# Gender differences in clinical characteristics and in-hospital and one-year outcomes of young patients with ST-segment elevation myocardial infarction under the age of 40

**DOI:** 10.34172/jcvtr.2021.17

**Published:** 2021-02-08

**Authors:** Bektas Murat, Eylem Kivanc, Rafet Dizman, Gurbet Ozge Mert, Selda Murat

**Affiliations:** ^1^Eskisehir City Hospital, Department of Cardiology, Eskisehir, Turkey; ^2^Eskisehir Yunus Emre State Hospital Department of Cardiology, Eskisehir, Turkey; ^3^Eskisehir Osmangazi University, Medical Faculty Department of Cardiology, Eskisehir, Turkey

**Keywords:** Acute Myocardial Infarction, Young Patients, Coronary Angiography

## Abstract

***Introduction:*** Although the incidence of acute ST-segment elevation myocardial infarction (STEMI) in the elderly population has decreased in recent years, this is not the case for young people. At the same time, no reduction in hospitalization rate after STEMI was shown in young people. Clinical characteristics, risk factors, angiographic findings, in-hospital and one-year outcomes of patients under the age of 40 and their gender differences were investigated.

***Methods:*** This study has been performed retrospectively in two centers. Between January 2015 and April 2019, 212 patients aged 18-40 years with STEMI and who underwent reperfusion therapy were included. The gender differences were compared.

***Results:*** The median age of (male 176; 83.0% and female 36; 17.0%) patients included in the study was 36 (33-38) for men and 36 (34-38) for women. Chest pain was the most common complaint for both genders (96.0% vs. 94.4%; *P* = 0.651). While men presented more often with Killip class 1,women presented more often with Killip class 2. The anterior myocardial infarction (MI) was the most common MI type and it was higher in women than in man (*P* = 0.027). At one year of follow-up, the prevalence of all-cause hospitalization was 24%, MI 3.8%, coronary angiography 15.1%, cardiovascular death 1.4%, and all-cause death 0.47%, there was no gender difference.

***Conclusion:*** Anterior MI was the most common type of MI and it was more common in women than in men. Left anterior descending artery was the most common involved coronary artery. The most common risk factor is smoking. In terms of in-hospital outcome, left ventricular ejection fraction was significantly lower in women. There was no significant difference in one-year outcomes between both genders.

## Introduction

Ischemic heart disease remains the leading cause of mortality and morbidity all over the world.^1^ Although acute ST-segment elevation myocardial infarction (STEMI) is mainly seen in the elderly, 4-10% of patients with acute STEMI have been reported to be under the age of 45.^2,3^ The incidence of STEMI in young individuals has been reported to be between 2-12% in different studies. This wide range of incidences depends on the use of different cut-off values ​​ranging from the age of 35-55 for the definition of ‘young’ in the studies.^4^ However, to the current ESC Guidelines, the term ‘young’ defines patients under 45, while those under 35 are ‘very young’.^5^ Although the incidence of acute STEMI in the elderly population has decreased in recent years, unfortunately this is not the case for young people and especially young women.^6^ Hospitalization did not decrease after acute STEMI in the young.^7^ The risk factors of young patients may differ from the elderly. The variability of coronary artery disease (CAD) risk factors between the young and the old has been demonstrated by various studies. In the young, acute STEMI is more common in men and the most common risk factors are smoking, family history and dyslipidemia.^2^ In some studies, it has been reported that acute STEMI has good short-term prognosis in young patients, though long-term prognosis is not.^8^ Some cohort studies have investigated the factors associated with an increased risk of mortality in young women with acute myocardial infarction. However, in most of these studies, the patients with STEMI and the ones with non-ST segment elevation myocardial infarction (NSTEMI) were both included, and age group identification was not homogeneous, and these studies lacked sufficient control group.^9,10^ Since young people are the most productive members of the society and the family, their exposure to serious ischemic heart disease such as STEMI engenders serious problems both psychosocially and economically. In this context, there is not enough information about clinical characteristics, risk factors, short-term and long-term mortality differences between both genders with acute STEMI at young age. In this study, we aimed to investigate the clinical characteristics, risk factors, angiographic findings, in-hospital and one-year mortality of patients under the age of 40, and their gender differences.

## Materials and Methods

**Figure 1:**
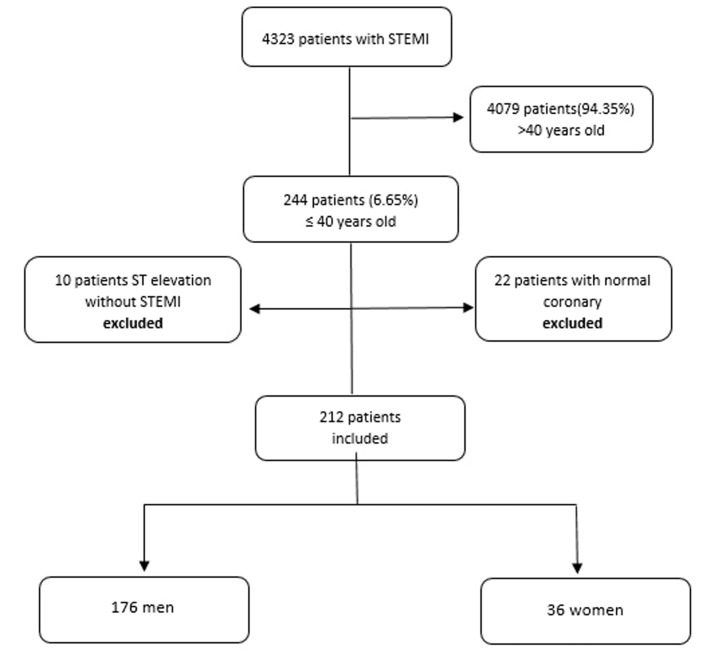
Flow chart of patient inclusion

This study has been performed retrospectively and observationally in two centers that provide 24/7 primary percutaneous coronary intervention (PCI) service. Between January 2015 and April 2019, 244 patients aged 18-40 years without previous coronary artery disease who presented to the emergency department with the diagnosis of acute STEMI and who underwent reperfusion treatment were included in the study. The patients who did not receive reperfusion therapy (primary PCI and/or fibrinolysis) were excluded. The patients whose coronary arteries were detected normal (n=22, female=13, male=9) in coronary angiography, and those with ST segment elevation excepting STEMI such as myocarditis and pericarditis (n=10) were excluded from the study. Eventually, 212 patients aged ≤ 40 years with critical coronary artery lesions (>50% left main coronary artery (LMCA) stenosis, and/or >70% any other coronary artery stenosis) who underwent PCI were included (Figure 1). The patients’ demographic information, age, gender, risk factors for coronary artery disease, and laboratory results were obtained from the data in the electronic systems of both hospitals. The patients were diagnosed with STEMI in conformity with Fourth Universal Definition of Myocardial Infarction Guidelines.^11^ The type and localization of myocardial infarction were determined through the electrocardiographic (ECG) findings. Patients with a body mass index >25 kg/m^2^ were defined as obese. Dyslipidemia was defined as serum total cholesterol (TC) ≥200 mg/dL, triglyceride (TG) >150 mg/dL, low-density lipoprotein (LDL) > 130 mg/dL, high-density lipoprotein (HDL) <50 mg/dL in women and <40 mg/dL in men, and/or those being treated with lipid-lowering therapy.^12^ As thrombus load was concerned, if the largest diameter of thrombosis was twice larger than the vessel’s one, it was defined as large thrombosis, otherwise small thrombosis.^13,14^ Reinfarction was defined as recurrence of ≥0.1 mV ST-segment elevation in at least two contiguous leads, or newly development of pathological Q waves in ECG; and rise of cardiac troponin (cTn) values with at least one value ≥20% of previous one, and the presence of symptoms suggestive of ischemia lasting 20 minutes or more.^9^ Stroke was defined as a focal neurological deficit lasting more than 24 hours due to an ischemic or hemorrhagic event, or a neurological event causing death.^12^ Major bleeding was defined as intracranial hemorrhage, cardiac tamponade, hemoglobin value falling more than 5 g/dL even if the bleeding focus could not be determined, and bleeding-related death. Any bleeding was defined by the TIMI criterion.^15^ The number of coronary artery involvement, the location of the intervention, interventional procedures and the treatments received by the patients, left ventricular ejection fraction (LVEF) before discharge, and the in-hospital results of the patients were obtained by retrospectively examining the electronic data systems of both hospitals. In-hospital results were comprised of data on pre-discharge EF value, reinfarction, cardiogenic shock, stroke, major bleeding, non-major bleeding, transfusion of blood and blood products, and all-cause mortality. The first-year follow-up of the patients was carried out by phone calls with them or their any relatives. The family history of CAD was questioned during the phone call and their first-degree relatives were taken into account. First year clinical results were comprised of hospitalization for any reason, myocardial infarction, coronary angiography (CAG), cardiovascular death and all-cause death (all death cause was defined as death from any cause other than cardiovascular disease). The study was approved by local ethical committee.

### Statistical analysis

Continuous data are given as medians (Q1-Q3). Categorical data are given as percentage (%). Shapiro Wilk’s test was used to investigate the suitability of the data for normal distribution. In order to compare the groups that do not conform with the normal distribution, Mann-Whitney U test was used for the situations with two groups. In the analysis of the created cross tables, Pearson Exact Chi-Square analysis was used. IBM SPSS Statistics 21.0 (IBM Corp. Released 2012. IBM SPSS Statistics for Windows, Version 21.0. Armonk, NY: IBM Corp.) program was used in the implementation of the analyses. For statistical significance, *P* < 0.05 value was accepted as criterion.

## Results

Two hundred forty four (5.65%) of 4323 patients who were admitted to the hospital with the diagnosis of acute STEMI and underwent reperfusion treatment were 40 years old and under. Among these patients, 22 were excluded from the study since their coronary arteries were normal. Besides, 10 of these patients were excluded, having ST-segment elevation apart from STEMI. The median age of 212 (male 176; 83.0% and female 36; 17.0%) patients included in the study was 36 (33-38) for men and 36 (34-38) for women. There was no difference between genders in terms of age. 13.7% of the patients were <30 years old and the youngest patient was 21 years old. Among the risk factors of coronary artery disease, the prevalence of smoking was 60%, dyslipidemia 31.1%, family history 16.6%, hypertension (HT) 15.1%, diabetes mellitus (DM) 14.2% and obesity 7.5%. Women were more obese than men (*P* = 0.008). While 95.5% of patients presented with typical chest pain and 18.4% with dyspnea, 12 patients had pre-hospital cardiac arrest. Although chest pain was the most common complaint for both genders (96.0% vs. 94.4%; *P* = 0.651), there was no statistically significant difference between the genders. Shortness of breath was more common in women (14.8% vs. 36.1%; *P* = 0.008). While men presented more often with Killip class 1, women presented more often with Killip class 2. When evaluated with regard to the type of myocardial infarction (MI), anterior MI the most frequent, inferior MI at the 2nd, and posterior MI at the 3rd frequent types in both genders, respectively. The most common MI was anterior MI in both genders. While there was no difference between genders in other MI types, anterior MI was more common in women than in men (75.0% vs. 55.1%; *P* = 0.027). It was observed that hematocrit and hemoglobin values ​were lower in women, however cTn and creatine kinase myocardial band (CK-MB) values ​​were higher (Table 1). Reperfusion therapy, the recommended treatment of acute STEMI, was applied to all patients, thrombolytic therapy was applied to one male patient, solely percutaneous transluminal coronary angioplasty (PTCA) to eight patients, and primary PCI to all other patients. Eight of the cases who had undergone primary PCI underwent coronary artery bypass graft (CABG) operation subsequently. There was no difference between genders in terms of reperfusion treatment types (*P* = 0.647). 95.3% femoral and 4.7% radial routes were preferred as the intervention site. When the number of vessels involved was evaluated angiographically, one-vessel involvement was 82.5%, two-vessel involvement was 9.9% and three-vessel involvement was 7.5%. The vessels involved were: the most common left anterior descending artery (LAD) 57.5%, right coronary artery (RCA) 13.7% as the second and circumflex coronary artery (CX) 9% the third. LAD was the most commonly involved coronary artery in both men (56.3%) and women (63.9%). Isolated left main coronary artery (LMCA) involvement was detected in two patients. There was no significant gender difference among the coronary angiography findings and the treatments administered in the first 24 hours (Table 2). In-hospital reinfarction was 2.8%, cardiogenic shock 1.9%, in-hospital death 1.4%, stroke 0.5%, major bleeding 0.5%, non-major bleeding 0.5%, and blood transfusion 0%, and there was no statistically difference between genders (Table 3). One of the cases deceased was female, two were male and the causes of death were cardiogenic shock.

**Table 1:** Baseline characteristics of female and male ST-segment elevation myocardial infarction patients ≤ 40 years old

**Baseline characteristics**	** Men** **(n = 176)**	**Women** **(n = 36)**	***P*** ** value**
Age, median (Q1-Q3), years	36 (33-38)	36 (34-38)	0.892
Age group, n (%)			
18-30 years	25 (14.2%)	4 (11.1%)	0.793
31-40 years	151 (85.8%)	32 (88.9%)	0.354
Risk factors of CAD, n (%)			
Family history of CAD	30 (17.2%)	3 (8.3%)	0.217
Cigarette smoking	107 (61.5%)	19 (52.8%)	0.354
Alcohol abuse	8 (4.6%)	3 (8.3%)	0.404
Addiction to drugs	2 (1.1%)	0 (0.0%)	1.000
Hypertension	26 (14.8%)	6 (16.7%)	0.799
Diabetes mellitus	25 (14.2%)	5 (13.9%)	1.000
Obesity	9 (5.1%)	7 (19.4%)	0.008
Dyslipidemia	56 (31.8%)	10 (27.8%)	0.697
Renal insufficiency	1 (0.6%)	0 (0.0%)	1.000
Clinical Features			
Typical chest pain, n (%)	169 (96.0%)	34 (94.4%)	0.651
Shortness of breath, n (%)	26 (14.8%)	13 (36.1%)	0.008
Out-of-hospital cardiac arrest, n (%)	10 (5.7%)	2 (5.6%)	1.000
Heart rate, median (Q1-Q3), bpm	78(72-91)	87(78-91)	0.070
Systolic blood pressure, median (Q1-Q3), mm Hg	120(110-120)	110(105-120)	0.080
Diastolic blood pressure, median (Q1-Q3), mm Hg	70(65-77)	70(60-71)	0.414
SpO_2_, median (Q1-Q3), %	100(99-100)	100(96-100)	0.056
Killip class on admission, n (%)			0.019
Class 1	155 (88.1%)	26 (72.2%)	
Class 2	13 (7.4%)	8 (22.2%)	
Class 3	5 (2.8%)	0 (0.0%)	
Class 4	3 (1.7%)	2 (5.6%)	
Type of myocardial infarction, n (%)			
Anterior myocardial infarction	97 (55.1%)	27 (75.0%)	0.027
Anterolateral myocardial infarction	7(4.0%)	1 (2.8%)	0.731
Inferior myocardial infarction	45 (25.6%)	6 (16.7%)	0.25
Posterior myocardial infarction	13 (7.4%)	1 (2.8%)	0.31
Lateral myocardial infarction	10 (5.7%)	1 (2.8%)	0.47
Inferolateral myocardial infarction	4(2.3%)	0 (0.0%)	0.36
Laboratory results on admission, median (Q1-Q3)			
Hemoglobin, g/dL	15.6(14.6-16.6)	14.7(13.0-15.8)	< 0.001
Neutrophil, 10^^3^/µL	7.04(5.25-10.43)	7.49(5.31-9.86)	0.890
Glucose, mg/dL	108(98-128)	112(99-143)	0.243
Creatinine, mg/dL	0.87(0.76-1.06)	0.79(0.69-096)	0.041
Total cholesterol, g/L	205(175-240)	202(170-225)	0.478
LDL cholesterol, g/L	123(99-151)	131(104-145)	0.821
HDL cholesterol, g/L	38.40(34.5-45.6)	43.5(38-45.7)	0.103
Triglycerides,g/L	162(111-263)	124(98-180)	0.114
Hs-Trp, pg/mL	170(36.1-360)	302(66.5-4299.7)	0.040
CKMB, ng/mL	6.9(1.55-17)	17.8(3.15-68.16)	0.028

Abbreviations: CAD, coronary artery disease; LDL, low-density lipoprotein; HDL, high-density lipoprotein; Hs-Trp, high sensitive troponin; CKMB, creatine kinase myocardial band

**Table 2:** Coronary angiography findings and cardiac procedures

**Coronary angiography findings, median (Q1-Q3)**	**Men** ** (n = 176)**	**Women** **(n = 36)**	***P*** ** value**
Acute reperfusion therapy			0.647
IV fibrinolysis	1 (0.6%)	0 (0.0%)	
Primary PCI	167 (95.4%)	35(94.6%)	
Procedure access, n (%)			0.069
Radial access	6 (3.4%)	4 (11.1%)	
Femoral access	170 (96.6%)	32 (88.9%)	
Number of diseased vessels, n (%)			
One vessel	146(83.0%)	29(80.6%)	0.730
Two vessels	17(9.7%)	4(11.1%)	0.790
Three vessels	13(7.4%)	3(8.3%)	0.845
Treated coronary arteries, n (%)			
Left anterior descending artery	99(56.3%)	23(63.9%)	0.390
Circumflex artery	18(10.2%)	1(2.8%)	0.150
Right coronary artery	25(14.2%)	4(11.1%)	0.620
Left main artery	1(0.6%)	1(2.8%)	0.210
The others	33(18.8%)	7(19.4%)	0.900
Thrombus burden, n (%)			
No thrombus	125 (71.0%)	26 (72.2%)	0.885
Moderate thrombus <2 diam.	36(20.5%)	7 (19.4%)	0.89
Large thrombus >2 diam.	15(8.5%)	3(8.3%)	0.97
Thrombus aspiration	15 (8.6%)	3 (8.3%)	1.000
Interventional therapy, n (%)			
PTCA	8 (4.6%)	0 (0.0%)	0.354
BMS	11 (6.9%)	2 (5.7%)	0.912
DES	140 (87.5%)	32 (91.4%)	0.854
No stent implantation	9 (5.6%)	1 (2.9%)	1.000
CABG	9 (5.2%)	3 (8.3%)	0.438
Medication received within first 24 hrs. n (%)			
Glycoprotein IIb/IIIa receptor antagonists	49 (28.7%)	12 (33.3%)	0.554
Aspirin	168 (99.4%)	36 (100.0%)	1.000
Statins	168 (99.4%)	33 (91.7%)	1.000
Beta-blockers	82 (48.8%)	17 (47.2%)	1.000
ACE inhibitors/angiotensin receptor II blockers	66 (38.6%)	16 (44.4%)	0.575
P2Y12 inhibitors	163 (95.3%)	35 (97.2%)	1.000
Low-molecular-weight heparin	161 (93.6%)	35 (97.2%)	0.696

Abbreviations:IV, intravenous;PCI, percutaneous coronary intervention; PTCA, percutaneous coronary angioplasty; BMS, bare metal stent; DES, drug eluting stent; CABG, coronary bypass graft surgery; ACE, angiotensin converting enzyme

**Table 3:** In hospital and one-year outcomes

**In-hospital outcomes**	**Men** **(n = 176)**	**Women** ** (n = 36)**	***P*** ** value**
LVEF at discharge, median (Q1-Q3), %	59 (50-61)	55 (45-60)	0.017
Reinfarction, n (%)	6 (3.4%)	0(0.0%)	0.592
Cardiogenic shock, n (%)	3 (1.7%)	1 (2.8%)	0.528
Stroke, n (%)	1 (0.6%)	0 (0.0%)	1.000
Major bleeding, n (%)	1 (0.6%)	0 (0.0%)	1.000
Any bleeding, n (%)	1 (0.6%)	0 (0.0%)	1.000
Blood transfusion, n (%)	0 (0.0%)	0 (0.0%)	-
Mortality, n (%)	2 (1.1%)	1 (2.8%)	0.429
One year follow-up outcomes			
Hospitalization, n (%)	42 (23.9%)	10 (27.8%)	0.672
Myocardial infarction, n (%)	7 (4.0%)	1 (2.8%)	1.000
Coronary angiography, n (%)	28 (16.0%)	4 (11.1%)	0.612
Cardiovascular death, n (%)	2 (1.1%)	1 (2.8%)	0.429
All-cause death, n (%)	1 (0.6%)	0 (0.0%)	1.000

Abbreviations: LVEF, left ventricular ejection fraction

Left ventricular ejection fraction before discharge was lower in women than in men (*P*= 0.017) (Table 3). Duration of hospital stay was an average of 4 days. There was no difference between genders with regards to duration of hospital stay.

During their one-year follow-up, four patients could not be reached either by phone or from the hospital records. All-cause hospitalization was 24%, MI 3.8%, CAG 15.1%, cardiovascular death 1.4%, and all-cause death 0.5%. One of the cases deceased due to cardiovascular reasons was a woman. When compared by gender, there was no statistically significant difference (Table 3).

## Discussion

The most important difference of this study from other ones is that all patients included in the study were ≤40 years of age with critical coronary lesions who underwent CAG for the first time with the diagnosis of acute STEMI. Among all patients who had acute STEMI in this study, 5.65% were in the age group of ≤ 40 years. The most common CAD risk factors were male gender, smoking, dyslipidemia and family history, respectively. Anterior MI was the most common type of myocardial infarction as localization, and single vessel involvement was most common.

Left anterior descending artery was the most common involved coronary artery among all in the course of infarction. Reinfarcts (2.8%), cardiogenic shock (1.9%) and in-hospital death (1.4%) were observed in the in-hospital clinical results. In the 1-year follow-up results, all-cause hospitalization was 24%, MI 3.8%, CAG 15.1%, cardiovascular death 1.4%. There was no difference between genders in terms of in-hospital and one-year follow-up results. Previous studies have suggested that acute MI is mostly seen in males in young patients.^3,16^ In our study too, 83% of all ≤40 years old patients were male and 17% were female, correlating with previous studies. This may result from the hormones such as estrogen protecting women from atherosclerosis, or it may be because of atypically symptomatic women. And lack of sufficient experience of the medical team that women can apply with the atypical and non-characteristic symptoms of acute MI can also lead to a missed diagnosis. There is not enough information about acute MI epidemiology in young people and there are serious differences of opinion in the definition of the term ‘young’ in the literature. Some studies have considered the age of <55 years as young, while others have considered the age of <40-45 years as young. In studies accepting the age limit as <40-45, the incidence of acute MI has been reported to be between 4-10%.^2,17^ ın our study, among all patients who had acute STEMI 5.65% were in the age group of ≤ 40 years. Since the first definition of acute MI, different inclusion criteria have been used in different geographies and different populations, especially in the last 10 years. Therefore, the incidence of acute myocardial infarction, gender distribution, atherosclerotic risk factors and clinical outcomes in the young may vary according to inclusion criteria. Although many risk factors have been identified in young patients with acute MI other than the traditional risk factors found in the elderly, at least one conventional cardiovascular risk factor is reported in most of these patients. In this study, smoking and dyslipidemia were the most common ones among traditional risk factors with 60% and 31%, respectively. Since more than half of the young patients who have had acute MI are already smokers, smoking keeps up being a major risk factor for acute MI in young adults, and more efforts should be made to reduce smoking in young people. There was no significant difference between genders in other traditional risk factors other than obesity (men were significantly higher than women, *P* = 0.008). In previous studies, inconsistent findings were reported on the main clinical characteristics of patients with acute MI and the variability of their clinical outcomes between genders. It is thought that the greatest reason for this discrepancy may be due to the different age limit of the patients included in the study and the inclusion of both STEMI and NSTEMI patient groups in most studies.^14,18-22^ Given the symptoms, the most common complaint of patients (95.5%) was typical chest pain. Although; typical chest pain was not statistically significant, it tended to be more common in men, while shortness of breath was significantly higher in women. Males applied mostly with Killip class 1 and females applied mostly with Killip class 2. In previous VIRGO (Variation in Recovery, Role of Gender on Outcome of Young AMI patients) and NRMI (National Registry of Myocardial Infarction) studies, too, it was reported that women applied with more atypical symptoms and therefore was late-diagnosed or misdiagnosed.^23,24^

One of the most important aspects of our study was that all STEMI patients included in the study underwent coronary angiography and reperfusion therapy. In previous studies, angiography in young MI patients revealed that 83% of the coronary arteries had critical lesions, and single vessel involvement was the most common finding. It has been reported that LAD was the most frequently affected coronary artery. When the number of vascular involvements was compared by gender, single vessel involvement was more common in women and multiple vessel involvement was more common in men.^20,25,26^ On the contrary, in recent studies, no significant difference was found between both genders in the number of vessel involvement. In the most recent study by Estelle et al., it was found that there was no difference between both genders in the number of vessel involvement.^14^ In our study, the most common type of MI was the anterior wall myocardial infarction, and the most common was single vessel involvement with regard to the number of coronary artery involvement. LAD involvement was the most common observed, among the coronary arteries. Although anterior MI was more common in women, there was no difference between the genders in terms of the number of vessels involved.

Unlike previous studies, reperfusion therapy was applied to all patients in this study. While only one patient was given thrombolytic therapy, 8 patients underwent CABG operation after primary PCI. There was no difference between the genders in patients undergoing reperfusion therapy type. Some previous studies reported that women applied to the hospital later, and therefore, women had less frequent reperfusion therapy. This situation is thought to be due to women having more atypical symptoms and being diagnosed later.^27^ In the VIRGO study, which is one of the largest studies conducted, it was revealed that women received less frequent and later reperfusion treatment than men.^28^ However, since only patients underwent reperfusion therapy were included in our study, the reperfusion therapy rate in all STEMI patients could not be determine and compared with the previous studies. In our study, creatine kinase-MB (CK-MB) and cardiac troponins (cTn) values were higher in women than in men in the blood tests performed at the time of admission to the hospital, besides, LVEF was significantly lower in women than in men in transthoracic echocardiography before discharge. The results of the current study are also an indication that women have applied to the hospital later in agreement with previous studies. However, contrary to these studies, the rate of women receiving reperfusion therapy was not different from men in our study. This situation may be interpreted as increasing awareness about the symptoms and risks of myocardial infarction among women over time.

Gender-based differences in acute MI management in the elderly are well known.^28,29^ However, gender-based differences in young people are limited to very few studies.^19^ Although some studies show that mortality, including both intra-hospital and post-MI 30 days, is higher in women than in men,^30^ there has been evidence that in recent years, mortality in young women have decreased and this has been linked to increased awareness and better risk profile.^31^ In a recent study conducted by Bandyopadhyay et al it was reported that in-hospital mortality did not differ between genders.^32^ In another study, he stated that, in-hospital results (except cardiogenic shock), especially mortality, did not differ significantly between genders.^14^ In our study, while in-hospital mortality was found for both sexes (1.4%) in ≤40 years old STEMI patients, no significant difference was found between genders. The most common cause of in-hospital death was found to be cardiogenic shock. Although no difference was found in our study, some studies reported that in-hospital post-PCI bleeding, reinfarction and cardiac arrhythmia were higher in women.^14,33^ It may be considered that these conditions may depend on cardiovascular risk factor profile, frequency of concomitant comorbid conditions, delay in reperfusion treatment, and whether effective treatment is used.^19,34^ Having a myocardial infarction at a young age has a great potential impact on mental health, ability to work, and socioeconomic status, as well as physical health and morbidity.

There are different data on the prognosis of acute MI in young people. Some studies have reported that although the early prognosis of STEMI is good in young people, the long-term prognosis is not different from the elderly.^8^ There is a study showing that long-term survival of young people with MI is better than older people.^35^ While some studies show that young women who have MI have a worse prognosis than men^31^; similar to the studies of Sweden,^36^ Norway^4^ and Korea,^37^ in our study, there was no significant difference in one-year mortality between men and women. In this study, there are some reasons why in-hospital and one-year results are different from previous studies, the most important of which is that the age range of patients in almost all previous studies was 45-60, and all MI types were included in the study. Our study was more homogeneous and the included patient group was ≤40 years old patients who underwent coronary angiography for the first time with the diagnosis of STEMI and who underwent reperfusion therapy.

Our study has some limitations. First, it was not possible to measure all clinical features of patients, as the study was observational and retrospective. Second, since the data was not available for all < 40 years patients presented with STEMI, the clinical profiles of patients not received reperfusion therapy in two centers might be different. Another limitation was that observation of the presence of spontaneous coronary dissection by optical coherence tomography or intravascular ultrasound method, the menopause status of women could not be learned and coagulation disorder by genetic tests in order to reveal the etiology of MI especially in the young was not performed. Another was that the number of men and women in the study was disproportionate in order to reveal the gender differences. And also Prospective randomized studies are needed to reveal exactly the incidence of MI, differences between genders and etiological causes in the young.

## Conclusion

In this study, which included patients under 40 years of age, Anterior MI was the most common type of MI as localization and it was more common in women than in men. LAD was the most common involved coronary artery in both genders. The most common risk factor was smoking in both genders. While the most common complaint of patients was typical chest pain, shortness of breath was significantly higher in women. In terms of in-hospital outcomes, LVEF was significantly lower in women than in men. There was no significant difference in one-year outcomes between both genders.

## Competing interests

The authors declare no conflict of interest.

## Ethical approval

This study was approved by ethics committee of Eskisehir Osmangazi University (12.05.2020-E.51611), with a waiver for informed consent because of the retrospective nature of the study.

## Funding

None.
